# Validity range of centrifuges for the regulation of nanomaterials: from classification to as-tested coronas

**DOI:** 10.1007/s11051-012-1300-z

**Published:** 2012-11-24

**Authors:** Wendel Wohlleben

**Affiliations:** BASF SE, Material Physics GMC/R, 67056 Ludwigshafen, Germany

**Keywords:** Nanoparticles, Size selective quantification, Characterisation for toxicology purposes, Analytical ultracentrifugation, Dynamic light scattering, Nanoparticle tracking, Hydrodynamic chromatography, Laser diffraction

## Abstract

Granulometry is the regulatory category where the differences between traditional materials and nanomaterials culminate. Reported herein is a careful validation of methods for the quantification of dispersability and size distribution in relevant media, and for the classification according to the EC nanodefinition recommendation. Suspension-based techniques can assess the nanodefinition only if the material in question is reasonably well dispersed. Using dispersed material of several chemical compositions (organic, metal, metal-oxide) as test cases we benchmark analytical ultracentrifugation (AUC), dynamic light scattering (DLS), hydrodynamic chromatography, nanoparticle tracking analysis (NTA) against the known content of bimodal suspensions in the commercially relevant range between 20 nm and a few microns. The results validate fractionating techniques, especially AUC, which successfully identifies any dispersed nanoparticle content from 14 to 99.9 nb% with less than 5 nb% deviation. In contrast, our screening casts severe doubt over the reliability of ensemble (scattering) techniques and highlights the potential of NTA to develop into a counting upgrade of DLS. The unique asset of centrifuges with interference, X-ray or absorption detectors—to quantify the dispersed solid content for each size interval from proteins over individualized nanoparticles up to agglomerates, while accounting for their loose packing—addresses also the adsorption/depletion of proteins and (de-)agglomeration of nanomaterials under cell culture conditions *as tested* for toxicological endpoints.

## Introduction

The existing regulatory framework for the registration, evaluation, authorisation and restriction of chemicals (REACH) does not contain specific provisions for nanomaterials. The commission’s scientific committees, European Food Safety Authority and Competent Authority Working Groups have confirmed that the established principles and approaches to risk assessment of substances are, in general, applicable to nanomaterials (SCENIHR 2009). Consequently, updates to the guidance documents on how to characterize nanomaterials have been drafted as appendix R7-1 (ECHA 2012) based on the scientific advice from the REACH Implementation Projects (RIPoN 2011). The Organization for Economic Cooperation and Development (OECD) judged only a minority of physico-chemical methods as directly applicable to nanomaterials, and for some additional properties that are required for nanomaterials, such as their state of agglomeration, OECD simply stated that ‘no easy methods exist’ (OECD 2009). But those materials that require nano-specific characterization first need to be identified amongst all existing particle-containing products (Calzolai et al. 2012): The European Commission recommendation for a regulatory nanodefinition classifies materials as nanomaterials if the number of particles with diameters below 100 nm exceeds 50 nb% of the total number of particles (% number per number) (EC 2011). Consequently, the entire size distribution must be determined in the number metric, which is irrelevant for application purposes and hence not recorded or specified. Particle-based products are contained primarily in paints, coatings and adhesives, and in bound state in many other products. Huge portfolios need to be screened from scratch, first for identification, then for nano-specific endpoints, such that methods applied must be accessible, cost-efficient and validated.

The very recent nano-specific guidance lists a significant number of techniques that may be adapted to satisfy the granulometric endpoints for specific materials: Optical microscopy, sieving, sedimentation, electrical sensing zone, phase Doppler anemometry, and especially for distributions extending below 100 nm: TEM, SEM, centrifugal sedimentation, ultrasonic spectroscopy, XRD, and DLS (ECHA 2012). The guidance articulates concerns on the validity of some of the most wide-spread ensemble methods such as DLS if the samples are polydisperse or in complex media: “…This method is of limited use when particles are difficult to maintain in a dispersed state or when particles of >2 μm in size are present. … DLS does not provide a full particle size distribution” (ECHA 2012). At the same time, the guidance puts emphasis on the characterization ‘as tested’ in physiological conditions, where nanoparticles are difficult or impossible to disperse due to aggregation/agglomeration processes. So what is the way forward? The present contribution aims to explore the ranges of validity of some techniques that are guidance-listed and/or well-established for the general characterization of these product classes—with a focus on fractionating methods. These are benchmarked against deliberately mixed and well-dispersed test samples of organic, metal-oxide, metal and carbon nanomaterials that have previously been thoroughly characterized at NIST or OECD level with specified properties. In this way, issues of sample preparation are circumvented in the interest of a reliable method validation. All methods discussed here depend on a near-perfect individualization of primary particles. This may not always be achievable for powders, which must then be assessed by a tool-box of methods (Calzolai et al. 2012).

For environmental or food samples, field-flow-fractionation (FFF) is probably the best developed technique (Klaine et al. 2012; Tiede et al. 2008). Several variants of FFF are known, with different detectors that are based either on the optical properties, on viscosity change or on elemental composition, and with different forces to establish the fractionation that the method carries in its name (von der Kammer et al. 2011). Mainly because of handling and because the upper resolvable diameters remain far below the micron range if sub-50-nm nanoparticles are to be detected (von der Kammer et al. 2011), FFF plays only a minor role in industrial product characterization and quality control.

Other fractionating techniques have developed into standards in those industrial branches that have a long experience with materials that may now fall into the range of the EC nanodefinition recommendation: For polymer dispersions, hydrodynamic chromatography (HDC) (Small 1974; Small and Langhorst 1982) is established even in high-throughput operation with 100 samples per day (Wohlleben and Schuch 2010).

For fine-size pigments, fillers and extenders in particular, centrifugation techniques complement the ensemble methods such as laser diffraction. Any analytical centrifuge uses synchronized detection systems to monitor a colloidal system *during* its fractionation by centrifugal forces. In contrast to HDC, there is a strong academic community driving the advancement of centrifuge techniques: Since its invention and validation on 21-nm gold nanoparticles (Svedberg and Rinde 1924) by Theodor Svedberg, rewarded with the Nobel Prize in 1925, applications with regard to protein association have dominated in academic research (Scott et al. 2005). Recently, however, numerous groups have reported decisive findings by centrifugation regarding the individualization, de-agglomeration and ligand adsorption of CNTs (Arnold et al. 2008; Backes et al. 2010a, b; Karabudak et al. 2010; Vankoningsloo et al. 2012); regarding as-tested nanomaterial size distribution and correlated protein corona of polymer particles (Walczyk et al. 2010) regarding shape (Zook et al. 2011), size (Roy et al. 2007), and corona (Jamison et al. 2008; Krpetic et al. 2011) of metals; regarding size and corona of metal-oxides in lung lavage (Schulze et al. 2011) or in serum components (Fabian et al. 2008; Landsiedel et al. 2010; Molina et al. 2011; Monopoli et al. 2011; Schäfer et al. 2012; Schulze et al. 2008). These innovations can be harvested to address the regulation of nanomaterials, and are put to test here.

On selected test cases, we also benchmark nanoparticle tracking analysis (NTA) (Carr et al. 2005; Filipe et al. 2010) due to its potential to directly assess number-based particle size distribution, and we supplement our results by a comparison to the wide-spread dynamic light scattering (DLS) and laser diffraction (LD) techniques. We compare techniques for plausibility and address primarily the size distribution and dispersability of as-produced nanomaterials, but also the applicability to ‘as-tested’ characterization of the changing state of agglomeration and adsorption events in a toxicological testing medium. Following this validation, we assess the reliability of different non-imaging methods to screen particulate materials for their potential regulatory classification as nanomaterial.

## Materials and methods

### Centrifuges (AUC)

#### Machines

**Fig. 1:**
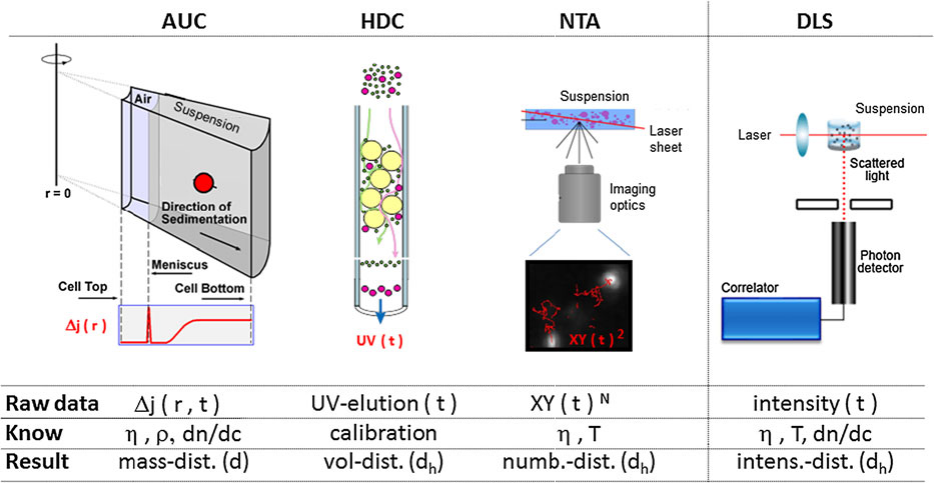
Principles of fractionating colloid characterization. (a) *AUC* analytical ultracentrifugation, (b) *HDC* hydrodynamic chromatography, (c) *NTA* nanoparticle tracking analysis (figure adapted from www.nanosight.com). Non-fractionating benchmark in (d) *DLS* dynamic light scattering (figure adapted from www.malvern.com)

In the shortest description, the analytical centrifuge uses synchronized detection systems to monitor a colloidal system *during* its fractionation by centrifugal forces (Fig. 1). A stunning variety of modes of operation has developed and is best described in the dedicated reviews (Cölfen and Wohlleben 2010; Mächtle and Börger 2006; Planken and Colfen 2010). The different commercially available geometries [disc (CPS, Brookhaven) vs. cuvette (LUM, Beckman)] do not compromise the comparability of results. All devices accept a wide range of solvents both in homogeneous and in line start (overlayering) sedimentation, except for CPS where differential line start sedimentation in an aqueous gradient is preferred (Riba et al. 2011). Standards were established for centrifuges (ISO 2001) with turbidity optics (ISO 2007) and for those with X-ray absorption optics (ISO 2004). While all other centrifuges require a ‘normal’ investment, comparable to, e.g., a DLS machine, the Beckman model XL-I costs a multiple, because it boasts of the highest centrifugal forces (a factor 10 above the fastest-spinning CPS machine) and of the interference optics, enabling the simultaneous colloidal characterization of particles and proteins or other macromolecules in a single experiment. Most of the results in this paper are obtained with the XLI, some with the turbidity-AUC or X-ray-AUC, but selected experiments in the present contribution and in literature (McFadyen and Fairhurst 1993) demonstrate that low-cost equipment such as the XDC (Brookhaven) performs just as well for most particulate characterization challenges.

As an academics-oriented alternative, the Open AUC Project provides workshop designs and (non-validated) operating software on an open-source basis (Cölfen et al. 2009; Strauss et al. 2008), and thus enabled a centrifuge that outperforms the Beckman benchmark (Backes et al. 2010a, b; Karabudak et al. 2009, 2010) but costs only fractions. In the same home-made way, BASF retrofitted a preparative Beckman centrifuge with turbidity optics (Mächtle 1984; Mächtle and Börger 2006) and Schlieren optics (Börger et al. 2004). Schlieren-AUC were commercial in previous times on Model-E and MOM centrifuges (discontinued), and are still the best optics for density gradients (Börger and Lechner 2006; Mächtle and Börger 2006; Mächtle and Lechner 2002). Turbidity-AUC is exactly identical to the Brookhaven DCP centrifuge in terms of the operating principle, detection and evaluation: They record turbidity at a specific distance from the center of rotation until particles pass by and turbidity drops. These procedures adhere to established standards (ISO 2001, 2007).

#### Standard evaluation

The commercial programs of turbidity centrifuges by Brookhaven and CPS, but also the programs for the turbidity optics integrated in Beckman centrifuges perform an iterative evaluation: First, one gets the diameter information from the measured time when the transmitted light intensity *I* = *I*(*t*) changes during constant or increasing speed of rotation; then one inverts the respective turbidity signal for each of these diameters via Mie theory to the mass-weighted size distribution (Lechner 2005; Lechner and Wohlleben 2008) The density *ρ*_S_ and viscosity *η*_S_ of the dispersing medium are required inputs. The ISO-standardized centrifuge methods then rely on the validity of the Stokes–Einstein equation

1 $$ d = \sqrt {\frac{{18 \cdot \eta_{s} \cdot S}}{{\rho_{\text{NP}} - \rho_{\text{S}} }}} , $$

where *s* is the material-specific sedimentation coefficient (Svedberg and Rinde 1924) which is the sedimentation speed *v* reduced by the machine-specific parameters of rotational frequency *ω* and distance *r* from the center of rotation to the detection position:

2 $$ S = \frac{\nu }{{\omega^{2} r}}. $$

The practical unit of *s* is 10^−13^ s. = 1 Sved(berg), with salts sedimenting below 1 Sved, proteins at a few Sved, organic nanoparticles around 100 Sved, inorganic nanoparticles at a few hundred Sved, agglomerates between 10^3^ and 10^6^ Sved. While in most cases, the density *ρ*_NP_ of the nanoparticles is a known input, it can also be determined in situ by differential sedimentation in normal and deuterated water, demonstrated for organic (Müller and Herrmann 1995), inorganic (Mittal and Lechner 2010), and adduct (Arnold et al. 2008) structures.

**Fig. 2:**
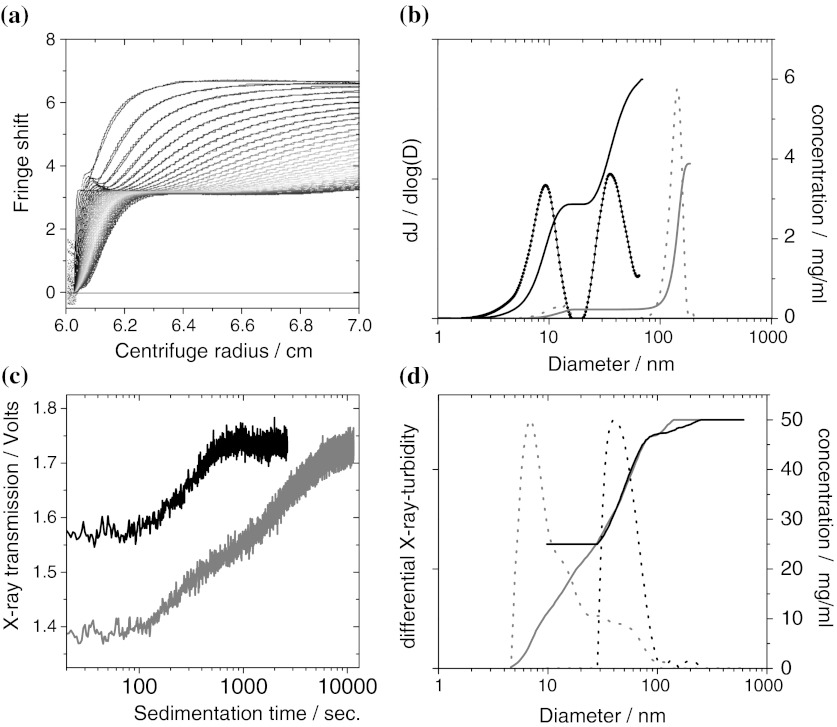
Validation of AUC on sub-100-nm SiO_2_ particles, showing raw data and evaluation for interference-AUC (*upper row*) and X-ray-AUC (*bottom row*). **a** Raw data and fit of interference-AUC on a 50/50 w/w mixture of Levasil 500 and Levasil 100, to highlight the direct reading of concentrations without any conversion. First scan at 162 s. after rotor acceleration, last scan at 2688 s., *rainbow color code* from *blue* to *red*. **b** Evaluation of the 50/50 w/w mixture of Levasil 500 and Levasil 100 (*black solid line* cumulative, *black dashed* differential) and of a 5 wt% spike of Levasil 300 with 95 wt% larger polymer beads (*grey solid line*: cumulative, *grey dashed* differential). The shares, the absolute concentrations and the diameters match the specified values. The signal from pure water is plotted, too (*thick black line*), but its noise level at 0.05 mg/mL is hardly visible. **c** Raw data of X-ray-AUC on a mixture 25 mg/mL Levasil 300 + 25 mg/mL Levasil 100 (*grey line*), and of 12 mg/mL Levasil 100 in the presence of 37 mg/mL polymer beads (*black line*). This highlights the selectivity of the X-ray-AUC for inorganic nanomaterial. **d** Evaluation of the X-ray-AUC raw data with the same *color code*. Note that the curve shapes are point-mirrored because larger particles sediment first and transformed by Eq. 1. (Color figure online)

The raw data from interference (absorption) optics gives a series of snapshots in time *t* of the radial profile of fringes (optical density, respectively) as shown in Figs. 1 and 2a. The entire dataset Δ*j* = Δ*j*(*r*,*t*) is evaluated by the free-ware software SedFit (Balbo et al. 2005; Schuck 2000). Alternatively, on may use the competing evaluation software package Ultrascan (Demeler 2005; Demeler et al. 2009). Over the centrifugation time scale of typically 1 h, diffusion becomes significant for small colloids around 10 nm diameter. The raw data from inteference optics must then be fitted with solutions of the Lamm differential equation, which describes the equilibrium of forces from centrifugal acceleration, from buoyancy and from friction, blurred by diffusion (Planken and Colfen 2010). Subsequently, the distribution in sedimentation coefficients s is transformed to a distribution in diameters *d* by the Stokes–Einstein equation (Eq. 1). The mass concentration shares *c* are read directly from the interference fringe shift Δ*j* with

3 $$ c = \frac{\lambda \cdot \Updelta j}{{{\text{d}}n/{\text{d}}c \cdot l}}, $$

where *l* = 12 mm the length of the optical cell, and *λ* = 675 nm the wavelength of the laser and d*n*/d*c* is the refractive index increment of the particular sample:

4 $$ {\text{d}}n/{\text{d}}c = \left( {n_{\text{NP}} - n_{\text{Solvent}} } \right)\frac{1}{{\rho_{\text{NP}} }} $$

Typical values for the parameters are *η* = 0.9333 g/m/s for DMEM, 0.9600 g/m/s for DMEM + 10 % FCS, 1.2003 g/m/s for FCS (all measured at 25 °C), and solvent density *ρ*_S_ = 1.0063 g/cm^2^ for DMEM, 1.0075 g/cm^2^ for DMEM + 10 % FCS, 1.0185 g/cm^2^ for FCS, all measured at 25 °C). DMEM is a cell culture buffer (Dulbecco’s modified Eagle medium), and FCS stands for Fetal Calf Serum.

#### Nanoparticle trace content detection limit

To define the detection limit of interference-AUC in the sub-100-nm region, we measured a water blank and obtained the curve shown in Fig. 2a, black line. With a refractive index increment d*n*/d*c* = 0.2 cm^2^/g, a typical value for inorganic nanomaterials, the integrated area under this curves gives a concentration of 0.05 mg/mL. This noise level defines the detection limit.

Note that the interference optics is strictly linear with the concentration, and no saturation occurs. The upper limit of measurable concentrations is defined by the spatial limits by the CCD imaging optics to resolve the fringe shifts and/or a turbidity above ~1 OD, because then the interference patterns is too low in contrast for the spatial Fourier transform algorithm that extracts the moving fringe shifts from the successive CCD snapshots. The maximal fringe shift around 100 fringes corresponds to 28 mg/mL (for a typical d*n*/d*c* = 0.2 cm^2^/g as above).

#### Evaluation adapted to nanoparticle fractal agglomerates

For well-dispersed primary particles (indexed ‘p’) with radius *r*_p_, one can rely on the Stokes–Einstein relation for spherical particles (Eq. 1) (Planken and Colfen 2010), but for agglomerates and aggregates, we have to consider their loose packing. With an additional particle attached to an agglomerate of *N* of primary particles, its hydrodynamic radius *R*_h,*N*_ grows stronger than for the addition of the same mass onto a solid sphere in form of a thin shell. Accordingly, these particle clusters must be described with a fractional dimension *D*_f_ < 3 (Balazy and Podgorski 2007; Limbach et al. 2005; Lin et al. 1990)

5 $$ N = \left( {\frac{{R_{{{\text{h}},N}} }}{{r_{\text{p}} }}} \right)^{{D_{\text{f}} }} $$

As limiting cases, *D*_f_ = 1 approaches the very unlikely morphology of a rod, and *D*_f_ = 3 describes a close-packed sphere. The following derivation of formula Eq. 7 is in line with standard references on the subject, such as Lin et al. (1990) except that many authors use the radius of gyration of the aggregate *R*_g,*N*_ for the calculation of the (obviously hydrodynamic) friction force (Balazy and Podgorski 2007; Limbach et al. 2005; Lin et al. 1990). *R*_h,*N*_ is typically larger than *R*_g,*N*_ due to the loose structure. The neglection of structure is not reasonable for a hydrodynamic method such as the AUC, but it seems to be indispensable for simplification of light scattering approaches.

For a *N*-agglomerate that sediments with velocity *v*_N_ as in Eq. 2, the forces from centrifugal acceleration *F*_sed,*N*_ and from friction *F*_f,*N*_ are in equilibrium:

6a $$ F_{{{\text{sed}},N}} = \sum\limits_{{}}^{N} {F_{{{\text{sed}},p}} = N\frac{4\pi }{3}\Updelta \rho r_{\text{p}}^{3} a} $$

6b $$ F_{{{\text{f}},N}} = 6\pi \eta \nu_{N} R_{{{\text{h}},N}} = 6\pi \eta s_{N} aR_{{{\text{h}},N}} $$

We finally obtain the relation between the radius of the agglomerate and its sedimentation coefficient *s*_*N*_ with due respect to the fractional dimension:

7a $$ s_{N} = \frac{2}{9}\,\frac{{N \cdot r_{\text{P}}^{3} \Updelta \rho }}{{\eta \cdot R_{{{\text{h}} .N}} }} = s_{p} \left( {\frac{{R_{{{\text{h,}}N}} }}{{r_{\text{P}} }}} \right)^{{D_{\text{f}} - 1}} $$

7b $$R_{{{\text{h,N}}}}  = R_{{{\text{h,N}}}} \left( {s_{N} } \right) = r_{{\text{p}}}  \cdot s_{{\text{p}}} ^{{\frac{1}{{1 - D_{f} }}}}  \cdot s_{{\text{N}}} ^{{\frac{1}{{D_{f}  - 1}}}} $$

Here *s*_p_ is the sedimentation coefficient of the primary particle. Previous reports did not detail the underlying assumptions of the derivation, but our Eq. 7b can easily be rearranged to match Eq. (6) in Lin et al. (1990). For practical use in AUC evaluation, the radius of the primary particles *r*_p_ must be determined independently, e.g., by TEM, and the fractional dimension *D*_f_ must be known. For reaction-limited colloidal agglomeration (RLCA), the value of *D*_f_ = 2.1 was measured for numerous materials (Lin et al. 1990) and applies universally, if collisions lead to agglomeration only with a certain probability, as is relevant for particulate suspensions (Evans and Wennerström 1994). The extracted radius scales then nearly linearly with the measured sedimentation coefficient $r \propto s^{0.9}$. Even smaller fractional dimensions (diffusion-limited colloidal aggregates, DLCA) lead to more-than-linear scaling of *R*_h,*N*_ with *s*_*N*_. In contrast, for *D*_f_ = 3 (solid particles), the resulting *R*_h,*N*_ is independent of *r*_p_ and Eq. 7 reduces to the classic Stokes–Einstein equation Eq. 1 with $r \propto \sqrt s$.

The standard evaluation hence significantly underestimates the size of agglomerates. By incorporating the fractional morphology into the AUC characterization of agglomerates, the retrieved diameters are corrected towards larger values. Note that even if the exact value of *D*_f_ may carry some uncertainty, a superposition of agglomerate signals with primary particle signals cannot occur. The validity of the enhanced AUC evaluation ceases if significant solvent flow through mesopores occurs or if shear forces induce structural changes, as was predicted for DNA (Schlagberger and Netz 2007). Both cases are not relevant for nanomaterial agglomerates.

### Hydrodynamic chromatography (HDC)

HDC in the sense of a packed column fractionating method for particulate systems has been introduced, investigated and baptized in 1974 by Hamish Small from Dow Chemicals (Small 1974; Small and Langhorst 1982). HDC specializes on “the discovery that the rate of transport of colloidal sized particles through a bed packed with *solid*, *non*-*porous* particles depends both on the particle size of the colloid and of the particles that constitute the packing” (Small 1974). The principle, performance, and limits of applicability were summarized in a recent book chapter (Wohlleben and Schuch 2010). In short, the separation column is made of non-porous cross-linked polystyrene beads with narrow size distribution around 15 μm, enabling a working range of 10–1,200 nm (Fig. 1). Due to the excluded streamline effect, all particles move 1–10 % more rapidly than the average flow speed of the carrier liquid. The entire time required for sample dilution in the elution buffer, for elution and evaluation is below 15 min, and the commercial HDC machines tolerate 14,000 polymer suspensions measured per year in our labs. Both diameter and peak width are deconvoluted with respect to calibration measurements, including Mie correction of shares derived from the UV-extinction at 254 nm (McGowan and Langhorst 1982; Williams et al. 2002). Applications beyond polymer particles include liposomes, silica, and gold/protein blends (Meehan and Tribe 2004). Here we use the commercial HD (PSDA by Polymer Labs, Agilent) without modifications and typical particle concentrations of 0.1–1 mg/mL (Wohlleben and Schuch 2010).

### Dynamic light scattering (DLS)

The temporal fluctuations of the scattered light intensity from an ensemble of suspended particles are characteristic of their average diameter (Fig. 1). To extract a distribution of diameters, the autocorrelation function of the scattered intensity is Laplace transformed to a distribution of diffusion times, and further to diameters via the known viscosity and temperature (Evans and Wennerström 1994). Mie correction is applied to the intensities via the known d*n*/d*c* in order to derive volume shares for each component. We used a HPPS (Malvern) with detection of back-scattered light at 173° scattering angle. Samples were diluted in Millipore water, and the absence of agglomeration was verified by the match of the measured diameter of the main peak with the specifications of the calibration particles. Evaluation was performed with contin-similar algorithm. If the size distribution showed a secondary component, we only required that it should be correctly above or correctly below the majority components, but it did not need to match exactly the known diameter. The share of that secondary or side component is reported by its weight content, even if the absolute diameter was failed.

### Fraunhofer laser diffraction

The size distribution of suspensions with diameters larger than 1 μm were measured by laser diffraction, which is based on the angular distribution of scattered light from an ensemble of suspended particles (Evans and Wennerström 1994). The d*n*/d*c* and absorption must be known to evaluate the size distribution based on fitting an overlay of characteristic diffraction patterns. We used a Malvern Master Sizer S with MS7-Cuvette Magnetically Stirred Cell. For TiO_2_, the tabulated refractive index of 2.8 was used. For CNTs, the imaginary refractive index was set to 1, and variations had little effect on the results. Samples at 0.5 mg/mL did not need to be diluted, but were in the admissible obscuration range between 2 and 10 %.

### Nanoparticle tracking analysis (NTA)

NTA records the two-dimensional projection of the diffusion path of suspended particles independently of each other. The viscosity and temperature of the medium must be known to extract a diffusion constant and hence diameter for each particle (Fig. 1). The histogram of particles sizes requires not further processing, specifically no Mie conversion, and is inherently counting. The NTA measurement device from NanoSight (LM20) has been thoroughly described and tested with bimodal mixtures of polymer calibration particles recently (Filipe et al. 2010). Previous evaluations revealed good applicability to phenomena relating to a time-dependent association starting from a known particle size, although concerns were raised on the inherent distribution narrowing algorithm due to the limited number of frames per particle (Montes-Burgos et al. 2010). The benchmark of NTA against DLS on bimodal distributions confirmed the superior tolerance of NTA against the presence of small amounts of large particles, but also reported imprecisions with regard to the total concentration depending on dilution steps (Filipe et al. 2010). To evaluate whether unknown samples with larger amounts side components of can be quantified, we followed their parameter range and adjusted the concentrations in dilution series to between 1 and 20 × 10^8^ particles/mL and used 25 frames/s, gain 5.5, and a minimum expected size of 30 nm.

### Reference and calibration particles

#### Polystyrene calibration particles, NIST-tracable

Here we used Duke Scientific Nanosphere Size Standards: Cat. No. 3050A, 46 nm ± 2 nm; Cat. No. 3100A, 97 nm ± 3 nm; Cat. No. 3400A, 404 nm ± 4 nm; Cat. No. 4009A, 993 nm ± 21 nm. The suspensions were diluted in the HDC elution medium for HDC, and in water for the other techniques. We did not perform additional characterization by TEM or otherwise, and instead rely on the specifications as certified.

#### Gold reference nanomaterials ex NIST

The reference materials “8011” and “8012” are citrate-stabilized Au nanoparticles in a aqueous suspension, sterilized by gamma irradiation. They were used as is without any further preparation or dilution. The effective concentration is not equal, but 51.56 μg/g (Au 8011) and 48.17 μg/g (Au 8012), respectively. The diameters were specified by NIST for multiple methods with the total uncertainty indicated:

**Table:** 

	Au 8011	Au 8012
AFM	8.5 nm ± 0.3 nm	24.9 nm ± 1.1 nm
SEM	9.9 nm ± 0.1 nm	26.9 nm ± 0.1 nm
TEM	8.9 nm ± 0.1 nm	27.6 nm ± 2.1 nm
DMA	11.3 nm ± 0.1 nm	28.4 nm ± 1.1 nm
SAXS	9.1 nm ± 1.8 nm	24.9 nm ± 1.2 nm
DLS	13.5 nm ± 0.1 nm	28.6 nm ± 0.9 nm^a^ 26.5 nm ± 3.6 nm^b^

^a^173° scattering angle, backscatter; ^b ^90° scattering angle

We did not duplicate any of the above characterization, and instead rely on the specifications as certified. It should be noted that the certificate reports also FFF results with gyration diameters of 11.5 nm and 28 nm, respectively.

#### Acrylic particles as challenge to universality of detection optics

Suspensions of acrylic latex particles (used for adhesives, paints, coatings) were taken from R&D at BASF. Their solid content is known to a precision better than 1 wt% (weight per weight) from the amount of monomer added during synthesis (ethylhexylacrylate and *n*-butylacrylate). The diameters were 70 and 390 nm. Compared to calibration latexes, which consist 99 % of styrene monomer units with optical resonance at 265 nm wavelength due to the benzene rings, the acrylic monomers allow little or no electrons delocalization, and hence optical resonances only at smaller wavelengths. We use the solid content as benchmark to compare the performance of different methods.

#### Silica particles, specified in solid content, and size

Amorphous SiO_2_ is produced in multi-kton amounts, both by gas-phase (pyrolytic) processes and by wet phase (sol–gel) synthesis. Here we used the very established product Levasil^®^ (HC Starck), delivered with high reproducibility as suspensions of individualized particles with anionic charge and solid content known to ±1 wt%. The product name indicates the BET surface area, and correspondingly diameters of 6–9–15–30–55 nm are specified for Levasil^®^ 500–300–200–100–50.

#### Agglomerated in situ dispersions of nanomaterials

We used materials from the OECD sponsorship program for nanomaterials, TiO_2_ (OECD NM105), mwCNT (OECD NM400), complemented by the same SiO_2_ (Levasil 200) as above. We did not perform additional characterization by TEM or otherwise, and instead rely on the multiply redundant results from OECD: TiO_2_ NM105 has a primary particle diameter of 21 nm (XRD, TEM), and specific surface of 51 m²/g (BET). CNT NM400 have an outer diameter of 9.5 nm and average length of 1.5 μm (TEM), and specific surface of 250 to 300 m²/g (BET).

The materials were dispersed at 1 mg/mL in H_2_O and probe-sonicated for 60 s. (Hielscher). The dispersion was then mixed with BSA in buffer, resulting in a final dispersion of 0.5 mg/mL nanomaterial and 1 mg/mL BSA in standard PBS. Due to the high specific surface of CNTs, a concentration of 5 mg/mL BSA was used for CNTs to keep the ratio of BSA mass per nanomaterial surface sufficiently high. The specific TiO_2_ and CNT are notoriously difficult to disperse in water. They were chosen not as ideal suspension (Bihari et al. 2008), but instead as typical representatives of ‘as tested’ suspensions with coexisting biological colloids (proteins), primary particles and agglomerates of the test material (Schulze et al. 2008).

## Results on classification of nanomaterials

### As-produced size distribution and dispersability

#### Size range 1–100 nm

As first benchmark, SiO_2_ nanoparticles with well-known diameter and concentration were measured individually. For a suspension of Levasil 500, we obtain 7.5-nm diameter and find 9.5 mg/mL, to be compared to specified value of 6 nm and the mass content of 10 mg/mL. The same level of accuracy is achieved when the sub-10-nm-SiO_2_ is to be determined in the presence of larger nanoparticles: Mixed with Levasil 100 at a mass ratio of 50 wt% (respectively, 5 wt%), the raw data is immediate evidence of the bimodality (Fig. 2a), and after evaluation we obtain for the minor component Levasil 500 indeed the correct diameter of 7 nm, shown as black line in Fig. 2b. Beyond size, the size-fractionated interference detection is a very precise concentration measurement, demonstrated here by the result of 48 wt% (6 wt%) for the minor component. Note that this result is independent of any corrections (such as Mie scattering in DLS) and in fact can be read directly from the raw data (compare Figs. 1, 2a).

In the next step, we simulate the presence of a minor inorganic nano-component in the presence of non-nano particles. We add 5 wt% (50 wt%) of Levasil 300 to a majority of polymer beads of 150 nm diameter. The larger particles sediment quickly and do not disturb the quantification of the sub-100-nm particles by interference-AUC. We obtain the correct share of 7 wt% (55 wt%) with the correct diameters for the SiO_2_ as minority component in the presence of the 150 nm particles (Fig. 2b).

In other cases, it may be very advantageous that the results from X-ray-AUC are completely independent of optical parameters. The X-rays are not tuned to a specific resonance, so that the raw data (Fig. 2c) is blind for organics, but selective for the inorganic material; this detection is not disturbed by optical turbidity, and scales directly linear with mass concentration. Applied to the same type of samples as above, the X-ray-AUC successfully distinguishes between 9 and 30-nm-SiO_2_ (Fig. 2d, black lines), and even quantifies the correct share of these components. Further, the X-ray-AUC successfully quantifies the diameters of SiO_2_ nanoparticles in the presence of larger polymer beads (Fig. 2d, grey line). The limit of detection is around 5 mg/mL solid content in the suspension, depending on the X-ray cross section of the actual inorganic material, as can be estimated from the signal-to-noise level of the raw data (Fig. 2c).

**Fig. 3:**
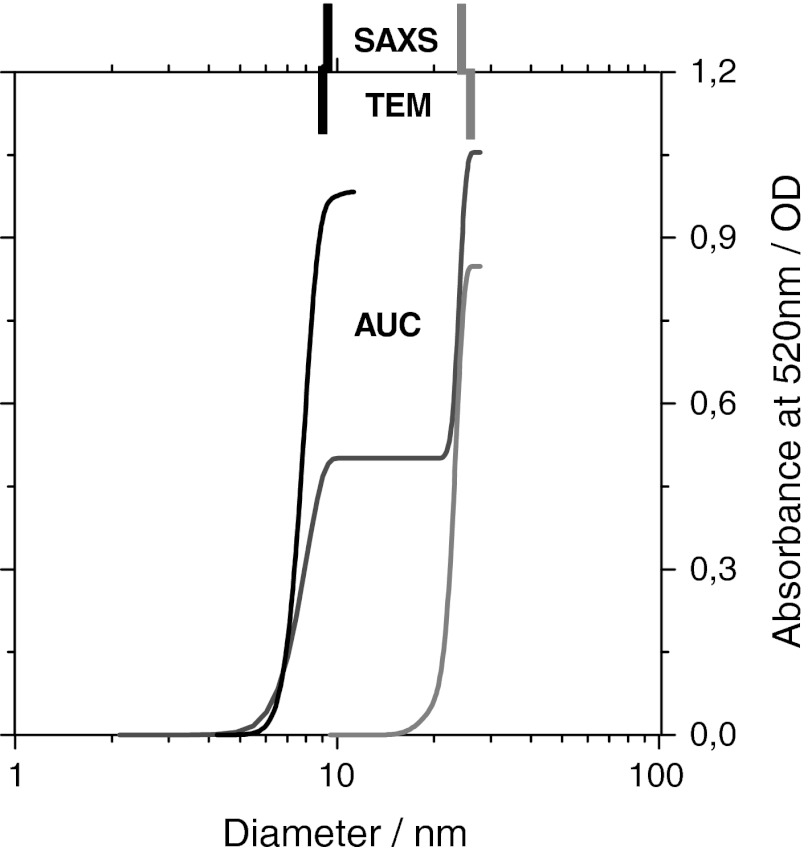
Analysis of NIST nano-gold reference materials. Cumulative size distribution of individual samples (Au 8011: *black line* measured at 5,000 rpm) (Au 8012: *grey line* measured at 3000 rpm) and their mixture (1:1 by suspension mass) (*red line* measured at 3,000 rpm). The specified values from SAXS and TEM are indicated as *tickmarks* in the respective *colors*. (Color figure online)

After metal oxide nanomaterials, the next important class is represented by metal nanomaterials, which are practically always supplied as suspension. Most metal nanomaterials are colored and thus offer a lever for selective detection by their characteristic absorption profile. Here, we employ the UVVIS-AUC for color movies during fractionation (Cölfen et al. 2009; Karabudak et al. 2010). Snapshots of absorption spectrum versus radial position are saved in 30-s time intervals during a 1-h sedimentation experiment. Beyond size, the UVVIS-AUC snapshots show correlations between size and color without further analysis, with obvious implications for plasmon adsorption phenomena (Zook et al. 2011). By selecting the wavelength of 520 nm for evaluation of sedimentation speed, we track selectively Au nanoparticles and quantify each fraction’s contribution to the optical density (right-hand axis in Fig. 3). We obtain a diameter of 8.1 nm (Reference Material 8011) and 24 nm (Reference Material 8012). These values are at the lower range of the other characterization methods, in good agreement with TEM, AFM, SAXS values, but significantly smaller than backscatter DLS. Using the bulk density of 19 g/cm^2^ for evaluation in Eq. 1, we neglect the citrate stabilization layer which is included by DLS. The agreement with alternative methods is excellent with 1 nm deviation for the larger species, where the relative contribution of the citrate stabilization layer to the total hydrodynamic diameter is negligible. The same effect was found in a round robin on SiO_2_ certification (Lamberty et al. 2011). An important asset of AUC is the measurement of the sizes in the mixture, where we find to less than 1 nm deviation for each fraction the same diameters as for the individual samples (Fig. 3, red line).

#### Sub-micron and nano size range with mixtures at 2–5–50 wt%

**Fig. 4:**
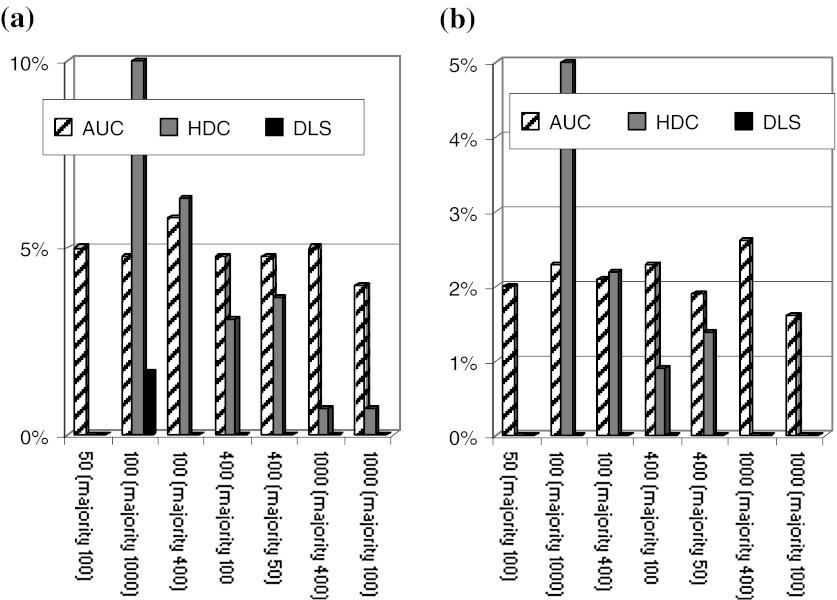
Shares of the side component detected on bimodal test cases of polystyrene calibration latexes. The *Y*-axis shows the share of the minor peak extracted from the entire size distribution, if there was a second peak above or below the majority component. The diameter of the admixture or minor component (in nm) is indicated on the *X*-axis, with the diameter (in nm) of the majority of particles in *brackets*. **a** admixture share of 5 wt%, **b** admixture share of 2 wt%

Due to the diameter range that is of interest here, turbidity-AUC in line with ISO (2007) was performed, but also interference-AUC would be applicable. Both were recently benchmarked for their accuracy with a round robin on monomodal nanomaterials (Lamberty et al. 2011). For our present investigations on multimodal distributions, NIST-traceable polymer bead standards were mixed as bimodal distributions. We added a side component of 2–98 wt% of a main component, where the wt% refers to the entire solid content of the sample. In Fig. 4, we indicate the diameter of the 2 wt% component (the diameter of the 98 wt% component in brackets). Both larger and smaller diameters are tested, and in total 47 bimodal samples were prepared and measured by three methods: DLS, HDC, and AUC.

The black bars in Fig. 4a show the amount of the side component detected by DLS—in fact, only one bar detaches from the baseline. DLS fails to tell us that either larger or smaller particles are present at the 5 % level.

With the equally fast technique HDC, the presence of the side component is detected in most cases (Fig. 4a, grey bars). This is an asset of the fractionating measurement principle, such that small and large diameters are detected independently of each other after their physical separation in the column. However, the HDC result is clearly not quantitative, with deviations up to a factor 4 or failed detection.

Quantitative analysis of bimodality is achieved with the AUC (Fig. 4a, hashed bars). For all test cases, the bimodality as such is unambigously detected, and the shares are correct with only 1 wt% deviation.

The same ranking of analytical methods emerges for an even lower share of 2 wt% side component. Again, DLS fails to detect that there is any bimodality (Fig. 4b, black bars), HDC does detect the bimodality in most cases, but with misleading shares (Fig. 4b, grey bars), and AUC achieves a quantitative result also on shares (Fig. 4b, hashed bars).

**Fig. 5:**
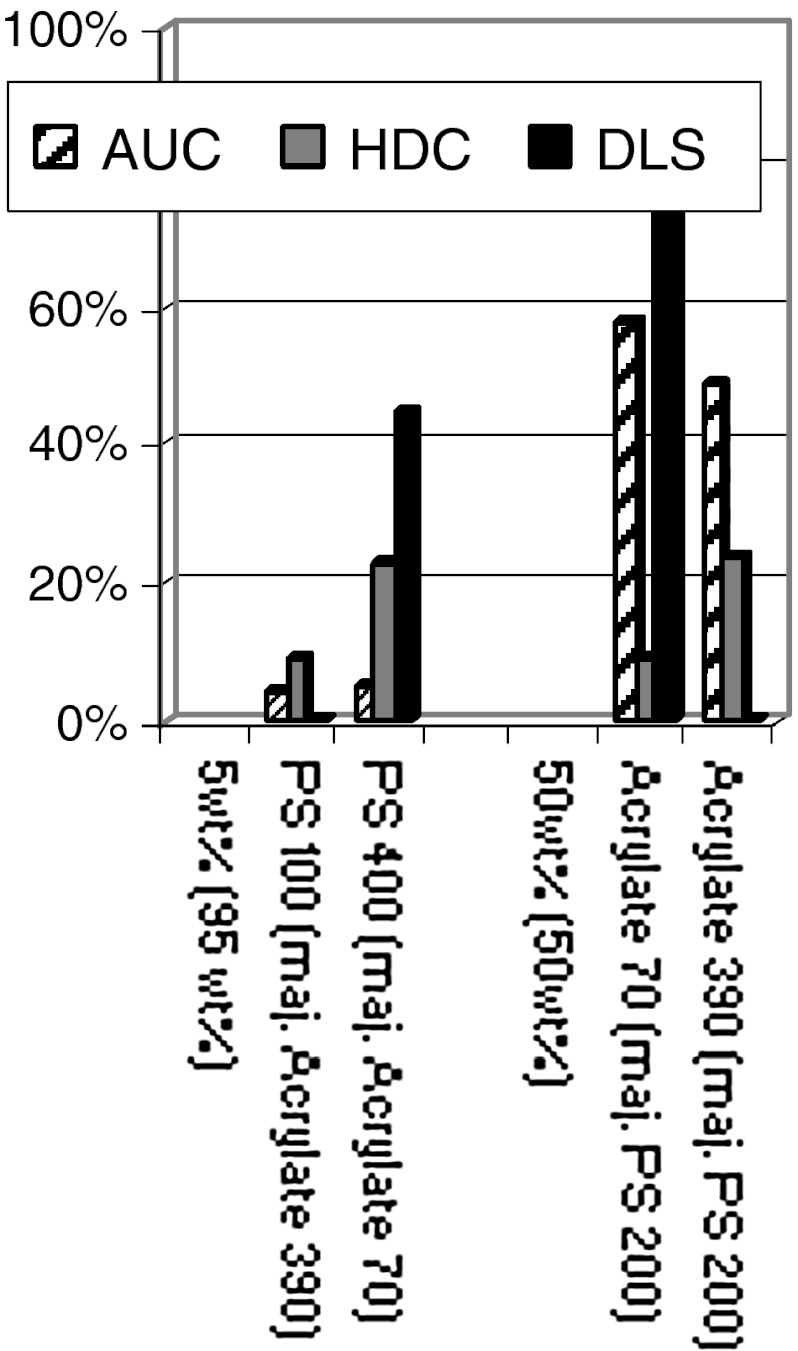
Shares of the side component detected on bimodal test cases of chemically inhomogeneous mixtures of styrenic (PS) and acrylate polymer particles. The *Y*-axis shows the share of the minor peak extracted from the entire size distribution, if there was a second peak above or below the majority component. The diameter of the admixture or minor component (in nm) is indicated on the *X*-axis, with the diameter (in nm) of the majority of particles in brackets. *Left rows* show results on share of 5**wt%** side component, *right row* with shares of 50/50 wt/wt mixtures

We now proceed to chemically inhomogeneous mixtures, first with 5 wt% styrenic particles within 95 wt% of acrylate particles. The deviations of DLS and HDC are even larger than for the same share of chemically identical particles (Fig. 5), and the deviations are also larger for turbidity-AUC, but still within a few percent of the real share. Deviations persist for 50/50 mixtures in all three methods, and can be attributed to the inhomogeneous optical resonances of the particles. Mie correction for the ensemble scattering (in DLS) and for the UV-turbidity (in HDC) is thus susceptible to false corrections. In the turbidity-AUC (in line with ISO13318) (ISO 2007) the resulting shares are still dependent on Mie correction, but the detection wavelengths are sufficiently far in the VIS (540 nm) that resonances do not dominate the signal.

**Fig. 6:**
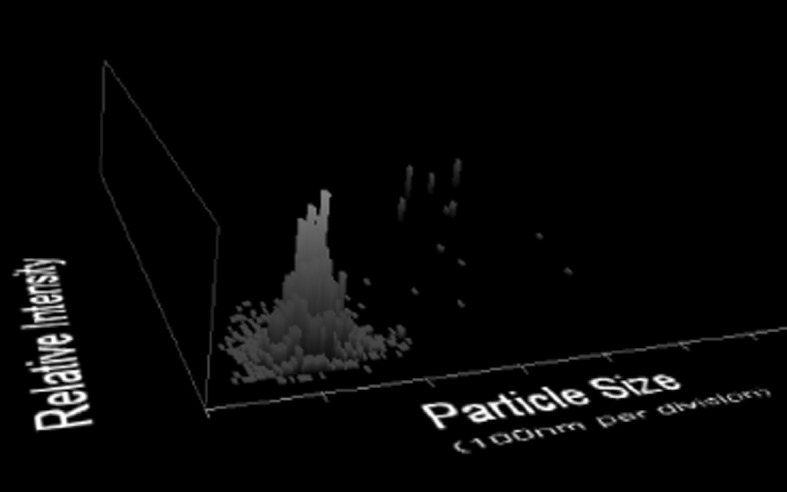
Nanoparticle tracking analysis, plot of relative intensity versus hydrodynamic diameter for the mixture of PS calibration latexes of 100 and 400 nm in ratio 95/5 wt%, corresponding to 99.9/0.1 nb%. A bimodality is detected successfully in this challenging case, although the retrieved share of 4 nb% at 400 nm deviates from the expectation

On a selection of the above test cases, we also applied NTA. In several cases, conditions of dilution and laser intensity could be found that allowed the detection of bimodality, e.g., for the 5 wt% minority of 100 nm in a 400 nm suspension (corresponding to ‘minority’ of 77 nb%) and vice versa with 400 nm as 5 wt% minority (0.1 nb%). NTA finds 35 nb% (for 100 nm as minority) and 4 nb% (Fig. 6, for 400 nm as minority), and hence performed significantly better than DLS for these cases. The absolute shares from NTA are quite impressive (in these two cases), considering uncertainties from number/mass conversion. Unfortunately in most other cases, e.g., mixes of 100 and 1,000 nm, or 50 and 400 nm, depending on light intensity either the large or the small particles were visible, but one could not easily detect the entire distribution. For the 73 nm acrylate with 200 nm PS, the resolution was insufficient to identify two peaks despite favorable number ratios.

## Discussion on classification of nanomaterials

To screen a large product portfolio requires a tiered approach to safe costs, and a tool-box of methods for different product classes (Calzolai et al. 2012). The following protocol is intended as Tier 1 classification, especially for materials that are supplied as suspensions, hence pre-dispersed. The Tier 1 approach requires a standardized protocol for sample preparation and a validated characterization technique. The EC recommendation focus on primary particles (EC 2011) necessitates the application of ultrasound to disrupt agglomerates beyond any naturally occurring shear force. A fractionating technique is then ideally situated to quantify minor components (as validated above), while maintaining an unrivaled statistical relevance: Starting with on the order of 10^11^ particles (200 μl measured sample volume at 0.1 mg/mL solid content), even the fractionated sub-ensembles represent on the order of 10^9^ particles, which is unreachable by imaging techniques. If more than 50 nb% of these particles range below 100 nm, the material would by classified as nanomaterial.

A major issue is that all established (standardized) non-imaging techniques with or without fractionation produce intrinsically vol.% or wt% data. If the shape of the size distribution can be trusted, then the distribution can be converted form vol.% or wt% to the regulatorily decisive nb%. However, false negatives are possible due to insufficient de-agglomeration or because a fraction below 100 nm is present, but below the detection threshold in wt% (typically between 0.1 and 10wt%). The conversion form wt% to nb% cannot correct this situation. In this case the material may still qualify as nanomaterial after more expensive Tier2 classification by, e.g., electron microscopy with image evaluation of at least 1,000 primary particles (for a 3 nb% confidence interval).

Note that 2 wt% (by weight) correspond already to 95 nb% (by number) for the case of a 99-nm side component in 1,000 nm main component, with increasing discrepancy for even smaller diameter contaminations. The detection limit within a size distribution hence must be on the 0.1 wt% level to extract trustworthy regulatory classification from wt% or vol.%-based size distributions.

The validation test cases (Figs. 4, 5) were designed before the publication of the EC recommendation, but cover the relevant range nonetheless. Eight of the bimodal distributions from Figs. 4 and 5 contain above 99 nb% below 100 nm and are correctly classified as nanomaterials by AUC (all results >95 nb%). The same holds true for the sub-100-nm mixtures from Fig. 2, which clearly qualify as nanomaterials.

The other six test cases are more intricate and are represented in terms of nb% in Table 1. The results in Figs. 4 and 5 demonstrate that non-fractionating techniques cannot be applied for regulatory classification (producing systematically false non-nano-classifications), whereas turbidity-AUC is valid for Tier 1 screening, and could be either the CPS or Brookhaven machines or retrofitted analogs. Note that for this measurement, a standard dilution of 4 mg/mL was chosen, such that the minor component was just above the detection limit. If the characterization task is to quantify exclusively the sub-100 nm trace components, on can measure at concentrations up to 50 mg/mL, where larger particles can still sediment without dragging the smaller particles away. One can then quantify the nanoparticles down to the detection limit which then corresponds to a share of 0.1 wt% of the solid content of the sample.

**Table 1:** Validation of regulatory classification by AUC with bimodal distributions (6 of 14 test cases)

Diameters of bimodal mixture (NM)	wt%	nb%	AUC nb%	Classification benchmark based on 50 nb% cutoff
50	5	30	30.5	Not nano: AUC OK
101	95	70	69.5	
99	5	98	97.9	Nano: AUC OK
1000	95	2	2.1	
99	5	77	80.2	Nano: AUC OK
400	95	23	19.8	
50	2	14	14.6	Not nano: AUC OK
101	98	86	85.4	
99	2	95	96	Nano: AUC OK
1000	98	5	4	
99	2	57	57.8	Nano: AUC OK (around threshold with AUC experimental error margins)
400	98	43	42.2	

The X-ray- AUC, considering its strict adherence to ISO 13381, considering further its commercial availability at investment costs below that of DLS machines, is another Tier 1 option. With an upper colloidal concentration around 100 mg/mL for unhindered sedimentation, the X-ray-AUC is not as sensitive as the turbidity-AUC or interference-AUC, but valid for down to 5 wt% minor components.

The recently introduced technique NTA (available from Nanosight or from Schaefer Tec) measures intrinsically number distributions, but is not standardized, especially not for the determination of nb% below a certain threshold. With NTA, we have adjusted parameters for optimum conditions, knowing the expected results. We would not have detected the bimodality with the same ‘blind routine approach’ that we took for DLS, HDC, and AUC. These findings are supported by a recent recommendation by the Environmental Protection Agency to use NTA for nanoparticle detection, but only when complemented by microscopic techniques (Jones-Lepp et al. 2011). Note the specific comment in the EC recommendation that the threshold is based on dividing the number of primary particles below 100 nm by the total number of primary particles. Hence, it is not sufficient to determine only the fraction below 100 nm, but the entire distribution is needed, which is a challenge for NTA.

Two other techniques were introduced very recently by university spin-offs that measure intrinsically number distributions (from Izon and from Affinity Biosensors). In the longer term, the hollow cantilever (Lee et al. 2010) has the potential to complement AUC with a counting approach using the same relations of size, mass and density. Whether broad distributions become measurable with the next generations of such apparatus needs to be evaluated with scrutiny, because larger particles may plug their sub-micron pores and hollow cantilevers. The Izon technique is a nanomaterial’s analog of the well-established electrical zone sensing, also known as Coulter counting, and duly mentioned in the REACH guidance draft (ECHA 2012). When passing through a tiny pore, a single nanoparticle reduces the ion countercurrent to a size-dependent extent (Kozak et al. 2011; 2012). That inherently counting size determination may require long measurement times and several pores to cover the entire size distribution, but first validation experiments against TEM and DLS were promising (Vogel et al. 2011).

In general, it would be naïve to define a single method for all aspects of the granulometry of nanomaterials, since the available methods report various relevant measurands with different metrics: with/without a solvent-swollen functionalization, with/without respect to agglomeration, number/mass/intensity distributions (Calzolai et al. 2012). The currently available nanoparticle-counting setups are too premature for regulatory use now, but we expect them to become part of the tiered approach after further operation refinement and validation.

Tiered approaches can safe enormous costs for the classification of large portfolios of particulate materials, because of lowered costs per measurement and enhanced accessibility of required equipment. The centrifuge results (Table 1) indicate that pre-dispersed materials can be indeed classified reliably as Tier 1 approach. For borderline cases, one would proceed to the Tier 2 classification by electron microscopy. Non-dispersable materials may rather use the volume-specific surface (VSSA) as proxy in Tier 1, and then proceed again to electron microscopy. Many, if not all powders currently have to be regarded as insufficiently dispersable, and the progress in methods needs to be accompanied by a significant progress in dispersion protocols and in simulations of the inherently agglomerate-tolerant conversion from mass to number distributions.

## Results and discussion on as-tested properties

### (De)agglomeration and adsorption by spontaneous bio-nano-hybridization

It is general wisdom that there is no such thing as a naked surface—and this holds arguably also for the large specific surface of nanomaterials (Grainger and Castner 2008). Already after production, organic contaminations are present on the surface (Landsiedel et al. 2010). But as soon as an inhaled nanomaterial lands on the lung lining fluid with its surfactant proteins and phospholipids, these naturally interface-active molecules will decorate the nanomaterial’s surface (Schulze et al. 2011). The same holds for the opsonization processes in serum, which have been investigated in detail for drug delivery purposes (Aggarwal et al. 2009). In the present contribution, we do not aim to address the influence of the protein corona (Cedervall et al. 2007) on recognition and fate. We restrict ourselves to the determination of the actual state of agglomeration of nanomaterials in physiological fluids. The challenge to characterization comes from the simultaneous polydispersities in chemical identity, morphology and diameter. Under realistic scenarios, the colloidal composition is dominated (both in mass and number) by proteins with diameters between 1 and 10 nm. The nanomaterial primary particles, if dispersed, are typically just above this diameter range, but lower in concentration. Nanomaterial agglomerates can reach several tens of micrometers.

**Fig. 7:**
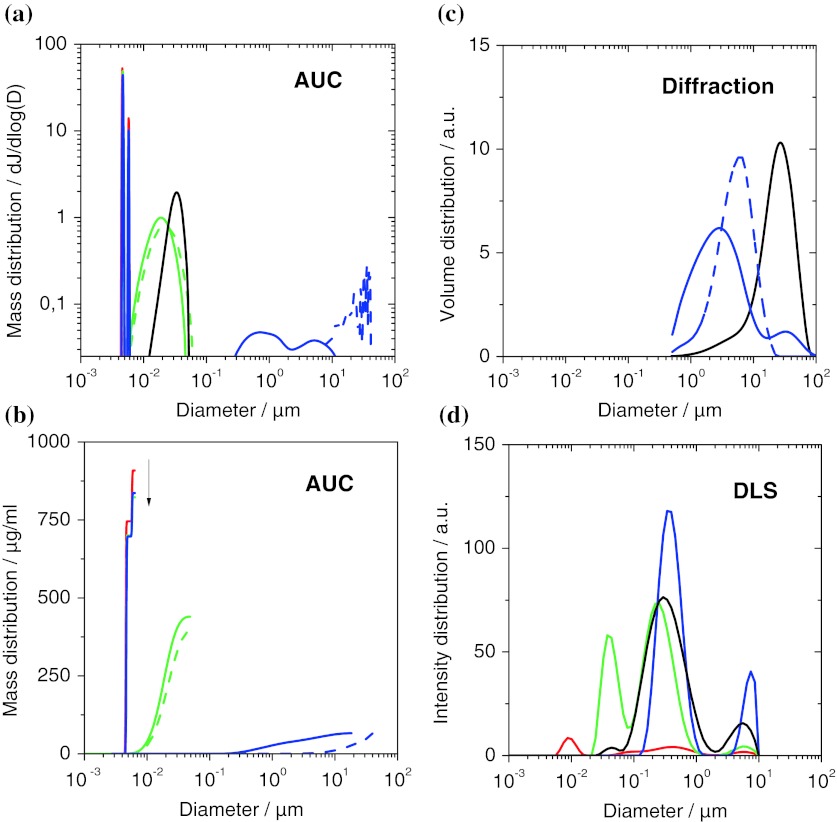
Comparison of in situ size distributions obtained on three representative materials: SiO_2_ (*green*), TiO_2_ (*blue*), mwCNT (*black*), pure PBS/BSA (*red*). **a** Differential representation of results from analytical ultracentrifugation (AUC). The peaks at 4.6 nm and 5.8 nm match the literature values of BSA monomer and dimer. *Dotted lines* indicate control experiments without BSA, leading to the disappearance of the peaks around 5 nm, and much stronger agglomeration of the TiO_2_ and CNT. **b** Cumulative representation of the same results to highlight the access to depletion data by quantifying the non-adsorbed proteins (*arrow*). Differential representation of results **c** from laser diffraction and **d** from dynamic light scattering (DLS). (Color figure online)

Based on the enhanced AUC evaluation with fractal dimensions of agglomerates as detailed in the methods, we characterized physiological suspensions of SiO_2_ (Levasil 200), TiO_2_ (NM105), and mwCNT (NM400) in PBS/BSA, benchmarked to the complementary methods of DLS and laser diffraction (Fig. 7). For DLS, the samples had to be diluted 10× in the buffer. DLS reports for all suspensions, including the empty buffer, a same central broad peak ranging from 100 to 1,000 nm (Fig. 7c). The suspensions differentiate by the appearance of additional peaks. Again, the agglomerate peak at 4 to 10 μm is universal in its reported position, which may be linked more to the technique than to the samples. The peak for the empty buffer at 10 nm does not match exactly the expectations for BSA, but can nonetheless be safely attributed to the protein. It is disconcerting to observe that this peak, although representing up to 90 wt% of the colloidal mass (and >99.99 nb%), is no longer detected in a nanomaterial suspension. If these 90 wt% at 10 nm are overlooked, why would one trust the signal at 20 nm to indicate the presence or absence of nanoparticles? This aspect adds to the failed validation of DLS *distributions* in Figs. 4 and 5. However, the DLS Z-*average* can be used to rank samples in the following order of increasing diameters: empty buffer, SiO_2_, mwCNT, TiO_2_.

Laser diffraction (Fig. 7d) catches only the agglomerates with a main peak from 500 nm to 20 μm and also finds that the diameters of TiO_2_ and CNT increase if the BSA is omitted (dashed lines).

AUC reports a very differentiated view of the colloidal diameter distribution (Fig. 7a). Two sharp peaks at 4.6 and 5.8 nm disappear if BSA is omitted (dotted lines), and vice versa they are the only remaining signals for the empty buffer (red line). Their position matches the literature values of BSA monomer and dimer, respectively, allowing a reliable attribution. The SiO_2_ is found as nearly individualized nanoparticles at 20 nm, and the mwCNTs appear at a diameter of 34 nm, which is just above their actual tube diameter.

For CNTs, the hydrodynamic size of a dispersed nanotube is close to its outer diameter, and is hence much smaller than the radius of gyration, which scales with fiber length, too. A quantitative agreement between AUC and laser diffraction cannot be expected for CNTs. The ranking by increasing diameter (empty buffer < SiO_2_ < mwCNT < TiO_2_) from AUC is hence in agreement with DLS. The TiO_2_, however, is found strongly agglomerated by AUC, and actually the cumulative representation (Fig. 7b) reveals that only 13 wt% of the TiO_2_ are within the detection interval. Using the enhanced evaluation with fractional dimension *D*_f_ = 2.1 for the TiO_2_, a quantitative agreement with laser diffraction for the agglomerate sizes is reached, both with and without BSA (Fig. 7d, a, respectively). In contrast, the minimal diameter increase of dispersed polymeric nanoparticles due to the adsorption of a BSA monolayer was seen by centrifuges first by us (Schulze et al. 2008), then confirmed and much developed independently (Walczyk et al. 2010; Monopoli et al. 2011).

But we can go further. The cumulative representation (Fig. 7b) gives us direct access to the mass concentration of all components, from the proteins to the dispersed nanoparticles and up to the agglomerates. We can directly observe the depletion of albumin from the water phase into the protein corona that then provides steric stabilization especially for mwCNTs, but to some extent also for TiO_2_ (Fig. 7b). Such indirect proof of a protein corona can be validated by, e.g., BCA protein assays for serum (Schäfer et al. 2012) or lung lining (Schulze et al. 2011) and has implications for mechanistic studies that go beyond regulatory purposes. To elucidate the presence and state of matter of nanomaterials that interact with complex media (physiological or food) it is mandatory to combine complementary measurement principles (imaging, fractionating, scattering) and assess the as-tested granulometry with multiple methods.

## Conclusion

Granulometry is the outstanding property where the differences between traditional materials and nanomaterials culminate (RIPoN 2011). Specifically for the endpoints (ECHA 2012) of size distribution (in relevant media) and for dispersability and for the classification according to the EC nanodefinition recommendation(EC 2011), we performed a careful validation: using test cases of several chemical compositions (organic, metal, metal-oxide), we benchmarked AUC, DLS, HDC, NTA against the known content of bimodal materials. The results validate fractionating techniques, especially AUC, which successfully identifies any nanoparticle content from 14 to 99 nb% with less than 5 nb% deviation. In contrast, our screening casts severe doubt over the reliability of ensemble techniques, especially DLS, which fails to detect the presence of nanoparticles even if these represent 99 nb% within 1 nb% of sub-micron particles. Finally the results indicate that NTA has the potential to develop within a few years into a ‘counting upgrade of DLS’, provided that the search space of dilution and light intensity is automated.

Contrary to pre-defined test cases, the true state of nanomaterials in relevant toxicological test media is not known beforehand. We addressed in situ adsorption, size distribution and agglomeration by benchmarking techniques against each other. The ranking of different nanomaterials in terms of their state of agglomeration is reproduced by both DLS and AUC, but only the enhanced AUC evaluation with fractional dimension of nanoparticle agglomerates reaches quantitative agreement for agglomerate sizes; the same measurement provides the absolute mass content of individualized and agglomerated nanomaterial as a measure of dispersability, and as a side product quantifies the depletion of albumins from serum onto the nanoparticle corona.
